# Eicosapentaenoic acid and docosapentaenoic acid monoglycerides are more potent than docosahexaenoic acid monoglyceride to resolve inflammation in a rheumatoid arthritis model

**DOI:** 10.1186/s13075-015-0653-y

**Published:** 2015-05-29

**Authors:** Caroline Morin, Pierre U Blier, Samuel Fortin

**Affiliations:** SCF Pharma, 235, route du Fleuve Ouest, Ste-Luce, QC G0K 1P0 Canada; Department of Pharmacology-Physiology, Faculty of Medicine and Health Sciences, Université de Sherbrooke, Sherbrooke, QC Canada; Department of Biology, Université du Québec à Rimouski, Rimouski, QC Canada

## Abstract

**Introduction:**

Rheumatoid arthritis (RA) is a chronic inflammatory autoimmune disease of the joints and bones. Omega-3 (ω3) fatty acid supplementation has been associated with a decreased production of inflammatory cytokines and eicosanoids involved in RA pathogenesis. The aim of this study was to determine the therapeutic potential of ω3 monoglyceride (MAG-ω3) compounds in an in vivo rat model of RA induced by Complete Freund’s Adjuvant (CFA).

**Method:**

CFA rats were untreated or treated per os with three specific compounds, namely, MAG-docosahexaenoic acid (MAG-DHA), MAG-eicosapentaenoic acid (MAG-EPA) and MAG-docosapentaenoic acid (MAG-DPA). Morphological and histological analyses, as well as pro-inflammatory marker levels were determined following MAG-ω3 treatments.

**Results:**

Morphological and histological analyses revealed that MAG-EPA and MAG-DPA exhibited strong activity in reducing the progression and severity of arthritic disease in CFA rats. Following MAG-EPA and MAG-DPA treatments, plasma levels of the pro-inflammatory cytokines; interleukin 17A (IL-17A), IL-1β, IL-6 and tumor necrosis factor α (TNFα) were markedly lower when compared to CFA-untreated rats. Results also revealed a decreased activation of p38 mitogen-activated protein kinases (p38 MAPK) and nuclear factor-kappa B (NFκB) pathways correlated with a reduced expression of TNFα, cyclooxygenase-2 (COX-2), matrix metalloproteinase-2 (MMP-2) and MMP-9 in paw homogenates derived from MAG-EPA and MAG-DPA-treated rats. Of interest, the combined treatment of MAG-EPA and vitamin E displayed an antagonistic effect on anti-inflammatory properties of MAG-EPA in CFA rats.

**Conclusion:**

Altogether, the present data suggest that MAG-EPA, without vitamin E, represents a new potential therapeutic strategy for resolving inflammation in arthritis.

## Introduction

The severity and disease progression of rheumatoid arthritis (RA) are governed by multiple factors including immune, genetic and environmental factors [1]. Joint lesions show infiltration of several immune cells including activated T lymphocytes, macrophages and antibody-secreting B lymphocytes into the synovium concomitant with a proliferation of synoviocyte cells [2, 3]. These latter cells together with new blood vessels form a tissue termed pannus, which leads to progressive destruction of cartilage and bone [2]. This phenomenon is most likely due to cytokine and eicosanoid-mediated induction of destructive enzymes such as matrix metalloproteinases (MMPs) [4]. Synovial fluid from patients with RA contains high levels of pro-inflammatory cytokines including TNF-α, IL-1β, IL-6, IL-8, IL-17A and granulocyte/macrophage colony stimulating factor (GMCFS) [5, 6]. Furthermore, both local and systemic levels of each cytokine are linked to disease severity [7–9]. Immune cells involved in RA usually contain a high proportion of the n-6 arachidonic acid (AA) and low proportions of other 20-carbon polyunsaturated fatty acids (PUFAs), with AA considered to be the major substrate for synthesis of eicosanoids [3]. Eicosanoids produced by both the cyclooxygenase (COX) and lipoxygenase (LOX) pathways are found in the synovial fluid of patients with active RA [10]. For example, expression of COX-2 is increased in the synovium of patients with RA and in joint tissues in rat models of arthritis [10, 11]. Protaglandin E2 (PGE2) and leukotriene B4 (LTB4), two eicosanoids respectively produced by COX and LOX, display a number of pro-inflammatory effects (including increasing vascular permeability), enhance local blood flow, are potent chemotactic agents for leukocytes, induce the release of lysosomal enzymes and enhance the release of reactive oxygen species and cytokines such as TNF-α, IL-1β and IL-6 [4, 10]. They also promote the production of destructive MMPs and stimulate bone resorption [2].

Nonsteroidal anti-inflammatory drugs (NSAIDs) are currently used to decrease pain and inflammation in RA patients [1, 12]. These agents exert their analgesic effects by inhibiting COX. Treatment of RA with NSAIDs, while improving symptoms, may lead to side effects such as gastrointestinal (GI) toxicity, osteoporosis, diabetes mellitus, weight gain, increased blood pressure, increased risk of heart failure and increased cardiovascular risk [3, 12]. As a result, these adverse effects have led to the restriction of NSAID use for the treatment of RA.

The dietary ω-3 PUFAs eicosapentaenoic acid (EPA) and docosahexaenoic acid (DHA), originating from fish oils, are also considered to reduce pain and inflammation in RA via the following mechanisms: ω-3 PUFAs competitively inhibit the production of PGE2 and LTB4, which in turn inhibit the activation of NFκB, and thus the release of inflammatory cytokines such as IL-1β and TNFα [13]. Moreover, ω-3 PUFA-derived mediators, including E-series resolvins (Rvs) such as RvE1 from EPA as well as D-series Rvs and protectin D1 from DHA, exert potent anti-inflammatory, inflammation resolving and immunomodulatory actions both *in vitro* and *in vivo* [14]. The ratio of ω6/ω3 PUFAs is important in RA pathogenesis with each of these acids differing in their efficacy. For example, EPA > DHA is effective against inflammation-induced arthritic markers in animal studies [15]. Studies using fish oil in patients with RA report decreased IL-1 production by monocytes [16] and decreased circulating concentrations of IL-1β, TNFα and soluble receptor activator of NFκB ligand [17, 18]. Moreover, clinical trials with ω3 PUFA supplementation have reported an improvement in the number of tender joints on physical examination, the Ritchie articular index, morning stiffness and decreased NSAID requirements [3, 19–25]. A meta-analysis of randomized controlled trials confirmed that ω-3 PUFA supplementation improves clinical symptoms of RA [26]. Moreover, a recent clinical trial demonstrated that fish oil used as adjunctive therapy in the context of modern treat-to-target drug treatment for recent onset RA both increased rates of remission and decreased drug use [27]. However, the conclusions of several clinical studies have shown consistent evidence for a modest clinical efficacy of marine ω3 PUFAs in RA.

In light of the above, EPA, DHA and docosapentaenoic acid (DPA) sn1-monoacylglycerides, namely eicosapentaenoic acid monoglyceride (MAG-EPA), docosahexaenoic acid monoglyceride (MAG-DHA) and docosapentaenoic acid monoglyceride (MAG-DPA), were synthesized in order to: 1) evaluate their effects on arthritic disease severity in a complete Freund’s adjuvant (CFA)-induced rat model, and 2) to monitor inflammatory arthritis activity using biochemical and histological analyses. Indeed, MAG-ω3 compounds are well-absorbed by the GI tract, are non-toxic, and their metabolites are found in blood circulation and tissues [28–31]. Specifically, we assessed the effects of treatment with MAG-ω3 compounds on morphological, clinical and histological features of arthritis disease. Moreover, the level of inflammatory markers (IL-17A, IL-1β, IL-6, TNFα, COX-2), the activation of p38 mitogen-activated protein kinase (MAPK) and NFκB pathways, as well as the levels of MMP2 and MMP-9 were determined following MAG-ω3 treatments. Results indicated that MAG-EPA and MAG-DPA exert more potent anti-inflammatory and pro-resolving effects than MAG-DHA in a CFA model of arthritis, a finding consistent with the inhibition of NFκB and p38MAPK pathways.

## Materials and methods

### Synthesis of ***ω***3 PUFA monoacylglycerides

MAG-DHA, MAG-EPA and MAG-DPA were synthesized as previously described [28, 29, 32].

### Animal model of arthritis

CFA was used to initiate induction of arthritis. Adult (10 weeks) female Lewis rats weighing 180 to 200 g were obtained from Charles River Laboratories (Montreal, QC, Canada). Rats were housed in our animal facilities in a 12:12-h light-dark cycle, at 22 ± 2 °C ambient temperature, and maintained on normal rodent chow and tap water *ad libitum*. Rats were acclimated 7 days before starting the experiments. All studies involving animals were approved by the institutional animal care committee of the *Université du Québec à Rimouski* (Protocol: # CPA-53-13-120). The rats were injected intradermally with 0.2 ml of CFA (Chondrex, Inc. Redmond, WA, USA) at the base of the tail. To increase the severity of arthritis, a booster injection with 0.1 ml of CFA was administered in the same manner on day 5. Measurements were obtained from both the inflamed and non-inflamed hind paws. The hind paw thickness (mm) was measured using a digital caliper. The severity of arthritis in the rats was assessed daily and scoring was attributed semiquantitatively (0: normal, with no macroscopic signs of arthritis; 1: mild, swelling and redness of one joint; 2: moderate, redness and swelling in two joints; 3: redness and swelling in more than two joints; 4: severe arthritis in multiple joints including the entire paw). Rats were randomly assigned into five groups: 1) control; 2) CFA; 3) CFA+ MAG-EPA-treated 4) CFA + MAG-DPA-treated, and 5) CFA + MAG-DHA-treated. MAG-ω3 compounds (318 mg/kg) were given orally directly to the back of the mouth with a pipette tip. MAG-ω3 treatments were administrated daily. The oral dose of 318 mg/kg was chosen according to Health Canada Draft Guidelines to obtain a human equivalent dose of 3.0 g/day (60 % of the maximum daily dose allowed by Health Canada) [33].

**Scheme 1:**
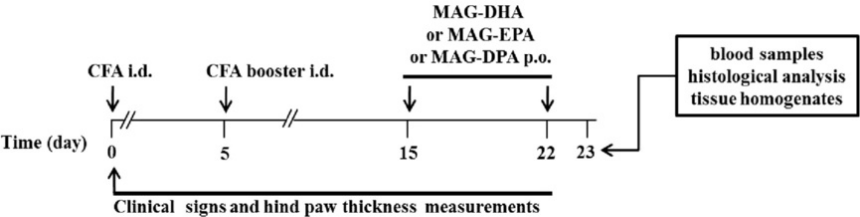
Experimental design and schedule of treatment in rat model of rheumatoid arthritis

Oral administration of MAG-ω3 compounds were initiated 15 days after the initial CFA injection and continued until study termination (day 22). Macroscopic signs of severe arthritis at 20 days included swelling, redness, deformity and ankyloses in the hind paw and ankle joints. At the end of the experiment, all five groups of rats were euthanized by a lethal dose of pentobarbital and blood and tissue samples were collected for further analyses (Scheme 1).

Additional experiments were performed to determine the effect of vitamin E and MAG-EPA treatments in CFA rats. MAG-EPA (318 mg/kg) and vitamin E (53 mg/kg) was given as a single dose orally directly to the back of the mouth with a pipette *tip*. Combined treatment was administrated daily 15 days following CFA injection after which its effects on hind paw thickness, COX-2 expression levels and pro-inflammatory cytokine profiles were evaluated.

### Western blot analysis

Western blots using specific antibodies against the phosphorylated forms of p65 NFκB (P-p65NFκB), p38MAPK (P-p38MAPK), as well as NFκB, p38 MAPK, COX2, TNFα, MMP-2 and MMP-9 and β-actin proteins were performed on hind paw homogenate fractions derived from control and CFA rats, either untreated or treated with MAG-EPA, MAG-DPA or MAG-DHA, as previously described. All antibodies used were obtained from New England BioLabs, Pickering, ON, Canada: 1 μg/ml of the selected specific antibody in TBS-T + 5 % BSA were incubated overnight at 4 °C. Immunostains of the blots were digitized and analyzed with Lab-Image software 2.7.

### Histological analysis

Rat tissues were fixed in 10 % buffered formalin and paraffin-embedded after which thin sections (3-μm thick) were stained with hematoxylin-eosin according to standard protocols [29]. Images were acquired with a Hamamatsu ORCA-ER digital camera attached to a Nikon Eclipse TE-2000 inverted microscope (Nikon-Canada, Mississauga, ON, Canada). Images were obtained (objective 20×) from hind paw sections derived from control, CFA, CFA + MAG-DHA-, CFA + MAG-EPA- and CFA + MAG-DPA-treated rats.

### ELISA assays

Measurements of key pro-inflammatory cytokines including IL-17A, IL-1β, IL-6 and TNFα were measured by specific ELISA on day 22 in plasma derived from control, CFA, CFA + MAG-DHA-, CFA + MAG-EPA- and CFA + MAG-DPA-treated rats, according to the manufacturer’s instructions (R&D Systems, Minneapolis, MN, USA).

### Data analysis and statistics

Results are expressed as means ± standard error of the mean (SEM), with n indicating the number of experiments. Statistical analyses were performed using Sigma Plot 11 and SPSS 14.0 (SPSS-Science, Chicago, IL, USA) using one-way analysis of variance (ANOVA) followed by Dunnett’s post-hoc test. Differences were considered statistically significant when *P* was <0.05.

## Results

### Effects of MAG-ω3 on arthritis severity

**Fig. 1:**
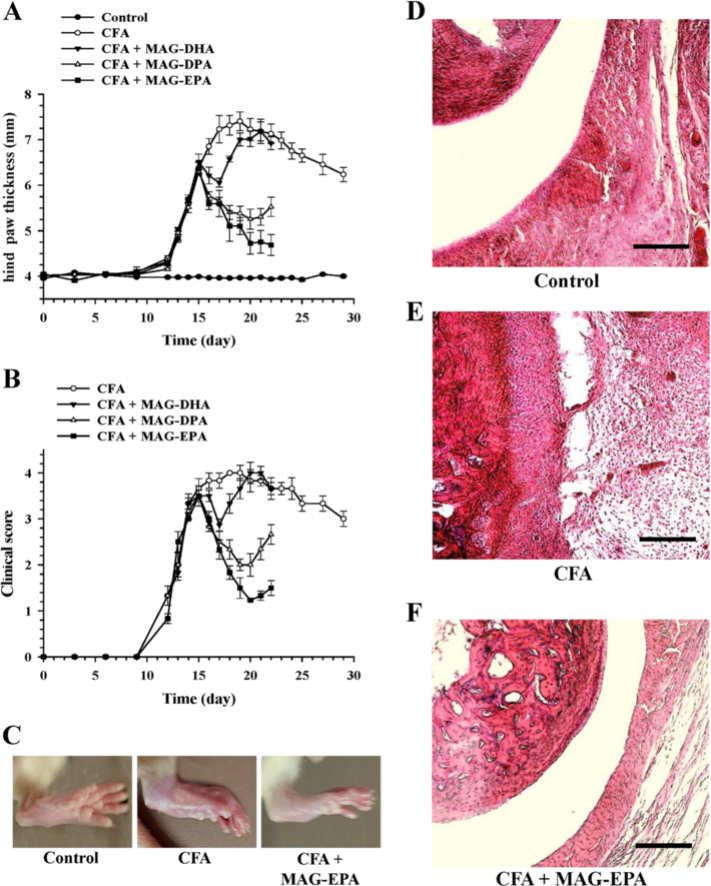
Effects of MAG-ω3 compounds on arthritis severity. **a** Hind paw thickness (mm) as a function of time (days) was measured in control and in complete Freund’s adjuvant (CFA) rats, either untreated or treated with docosahexaenoic acid monoglyceride (MAG-DHA), eicosapentaenoic acid monoglyceride (MAG-EPA) or docosapentaenoic acid monoglyceride (MAG-DPA) (318 mg/kg). MAG-ω3 compounds were administered at 15 days post-CFA injection onward. Six control and six CFA rats were sacrificed on day 22 at the same time as CFA + MAG-ω3-treated rats for pro-inflammatory marker analyses, whereas six controls and six CFA rats were sacrificed on day 29 to evaluate the progression of arthritis over time. Results represent the mean ± standard error of the mean (n = 6 per group for MAG-ω3-treated animals and n = 12 for control and CFA rats). **b** Clinical arthritis score as a function of time was determined in untreated and treated CFA rats (n = 12 for untreated and n = 6 for treated conditions). **c** Macroscopic images of hind paw derived from control, CFA and CFA+MAG-EPA-treated rats. Hematoxylin-eosin staining of hind paw thin sections derived from Control (**d**): CFA (**e**) and CFA + MAG-EPA-treated animals (**f**). Bar = 50 μm

The anti-inflammatory activity of MAG-DHA, MAG-EPA and MAG-DPA was assessed in a rat CFA model, a widely-used model for human RA. Figure 1a illustrates hind paw diameter measurements from control, CFA, CFA + MAG-DHA-treated, CFA + MAG-EPA-treated and CFA + MAG-DPA-treated rats. Results indicate that 18 days following initial CFA injection, hind paw thickness of CFA-untreated rats was significantly increased (7.32 ± 0.22 mm) when compared to control rats (3.96 ± 0.01 mm, Fig. 1a). MAG-DHA treatment of CFA rats resulted in a transient reduction in hind paw thickness following the first and second treatment day, whereas on day 18 to 22, hind paw thickness measurements increased to reach the same level as that of CFA-untreated rats (Fig. 1a). In contrast, treatment of CFA rats with MAG-DPA and MAG-EPA resulted in a significant reduction in hind paw thickness when compared to untreated CFA rats (Fig. 1a). Moreover, data indicated that MAG-EPA displayed a more potent effect on the reduction of hind paw swelling and redness on day 20 to 22 than MAG-DPA treatment (Fig. 1a). The arthritic index represents the grade of arthritis that was used to assess the efficacy of MAG-ω3 compounds. In the CFA group, diseased rats without any treatment showed an increased arthritic index starting on day 10 to a peak on day 18 (Fig. 1b). Compared with CFA group, administration of MAG-DHA did not significantly reduce arthritis score (Fig. 1b). However, MAG-EPA and MAG-DPA were observed to have significant activity in preventing the progression of arthritic disease with the arthritis score significantly lowered in treated CFA rats from day 16 to 22 (Fig. 1b).

Moreover, as illustrated in Fig. 1c, macroscopic images revealed that MAG-EPA-treatment decreased arthritic symptoms when compared to those observed in the untreated CFA group. Histological analysis of joints in CFA rats demonstrated an extensive proliferation of synovial cells, resulting in pannus formation and infiltration of leukocytes to the sub synovial region, with damage to articular surfaces and discontinuity in the cartilage when compared to histological joint sections obtained from control rats (Fig. 1d-e). However, CFA rats treated with MAG-EPA showed a reduced level of severe arthritic and degenerative changes when compared to the morphological changes observed in the untreated CFA group (Fig. 1f).

### Effects of MAG-ω3 on pro-inflammatory cytokine levels

**Fig. 2:**
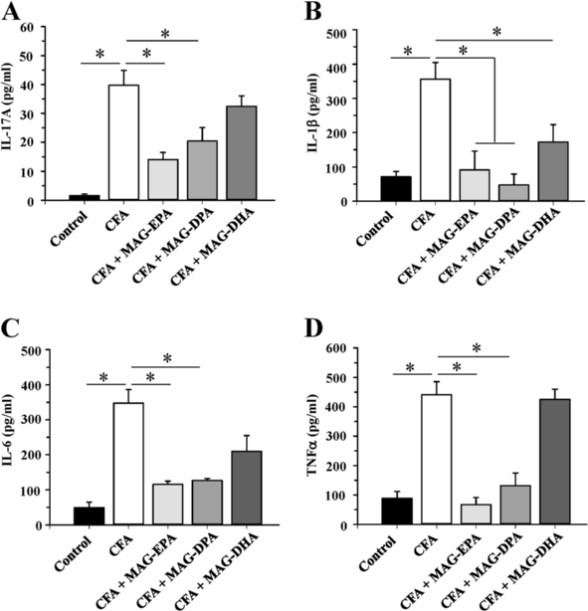
Effects of MAG- ω3 treatments on pro-inflammatory cytokine levels. (**a**) IL-17A, (**b**) IL-1β, (**c**) IL-6 and (**d**) TNFα levels were assessed in plasma of control, complete Freund’s adjuvant (CFA)-treated, CFA + eicosapentaenoic acid monoglyceride (MAG-EPA)-treated, CFA + docosahexaenoic acid monoglyceride (MAG-DHA)-treated and CFA + -docosapentaenoic acid monoglyceride (MAG-DPA)-treated rats using specific ELISA as described in the Methods section (n = 6, *P ≤0.05)

To investigate possible mechanisms by which MAG-ω3 compounds decrease arthritis progression, levels of key pro-inflammatory cytokines including IL-17A, IL-1β, IL-6 and TNFα were measured by specific ELISA on day 22 in plasma derived from control, CFA, CFA + MAG-DHA-, CFA + MAG-EPA- and CFA + MAG-DPA-treated rats. As illustrated in Fig. 2, IL-17A, IL-1β, IL-6 and TNFα levels were significantly higher in the plasma of CFA rats when compared to plasma levels in control animals. In contrast, the levels of these cytokines were significantly lower in the plasma of MAG-EPA- and MAG-DPA-treated animals when compared to untreated CFA rats. Of note, circulating levels of IL-17A, IL-1β, IL-6 and TNFα were less reduced in the presence of MAG-DHA than in MAG-EPA- and MAG-DPA-treated animals.

### Effect of MAG-ω3 on NFκB pathway activation

**Fig. 3:**
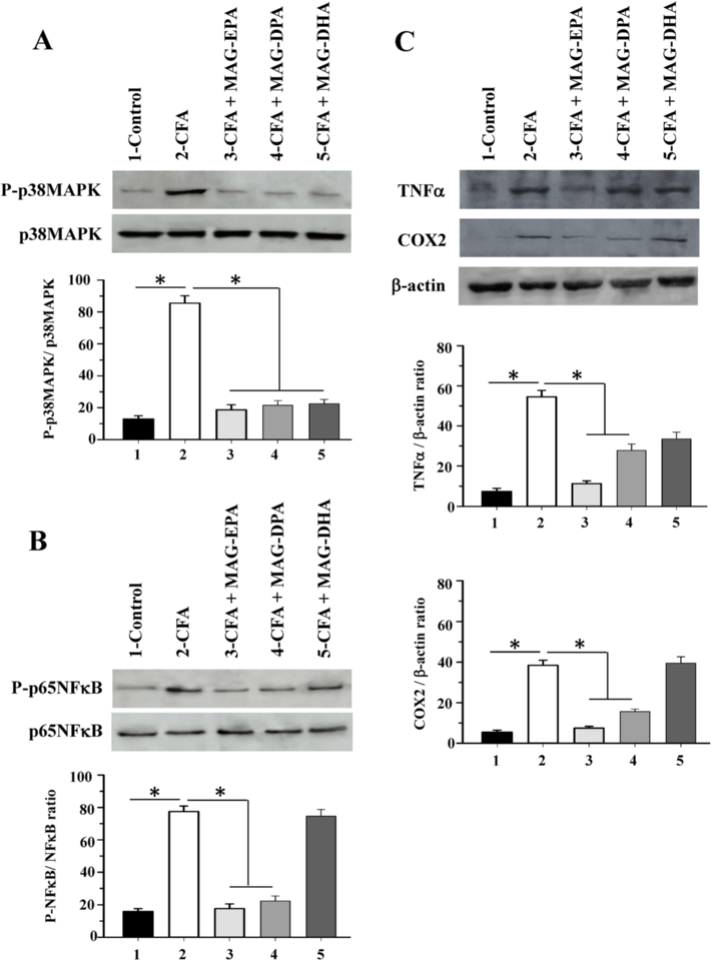
Effect of MAG-ω3 on activation of p38 mitogen-activated protein kinase (MAPK) and NFκB pathways. **a** Western blot and quantitative analysis of hind paw homogenates derived from control complete Freund’s adjuvant (CFA), CFA + eicosapentaenoic acid monoglyceride (MAG-EPA), CFA + docosapentaenoic acid monoglyceride (MAG-DPA) and CFA + docosahexaenoic acid monoglyceride (MAG-DHA)-treated rats using specific antibodies against the phosphorylated form of p38MAPK (P-p38MAPK) and total form of p38MAPK. Staining densities of P-p38MAPK in homogenates are expressed as a function of p38 MAPK signals (n = 6, *P <0.05). **b** Western blot analysis of the phosphorylated form of p65 NFκB and total form of p65 NFκB in control, untreated CFA and CFA + MAG-ω3- treated rats. Staining densities in paw homogenates are expressed as a function of total p65 NFκB (n = 6, *P <0.05). **c** Western blot and quantitative analyses of TNFα, cyclooxygenase (COX)-2, and β-actin protein detection derived from control and the four series of CFA-treated rats. Staining densities in homogenates are expressed as a function of β-actin signals. (n = 6, *P <0.05)

P38 MAPK and NFκB pathways are known to contribute to the overexpression of pro-inflammatory cytokines, chemokines, MMPs and signaling enzymes such as COX-2 in the inflamed synovium. In order to determine whether the above anti-inflammatory effects of MAG-ω3 compounds are mediated by these specific pathways, the activation of p38MAPK and NFκB was investigated by western blot in paw homogenates derived from control and CFA rats, either untreated or treated with MAG-EPA, MAG-DPA or MAG-DHA. Western blot analysis revealed that MAG-EPA, MAG-DPA and MAG-DHA treatments all decreased CFA-induced phosphorylation of p38 MAPK to a similar extent compared to untreated CFA animals (Fig. 3a). Western blot and quantitative immunoblot analyses were also performed on hind paw homogenates derived from untreated control and untreated and treated CFA rats using antibodies against total and phosphorylated forms of p65 NFκB. Results revealed that MAG-EPA and MAG-DPA treatments decreased p65 NFκB phosphorylation levels in paw tissues compared to that observed in untreated CFA rats (Fig. 3b), with significant reductions of 81 ± 3.1 % and 76 ± 2.7 %, respectively, following comparative analysis of P-NFκB/NFκB ratios after normalization of identical immunoblot membrane areas (Fig. 5b). However, no significant difference in p65 NFκB phosphorylation levels in CFA + MAG-DHA-treated rats was observed when compared to untreated CFA animals (Fig. 3b).

We assessed the expression of TNFα and COX-2 in tissue homogenates derived from control as well as untreated and MAG-ω3-treated CFA rats. Results revealed a significant increase in TNFα and COX-2 protein expression in CFA rats when compared to expression levels in control animals. Treatment with MAG-EPA and MAG-DPA, however, reduced TNFα and COX-2 expression levels compared to untreated CFA animals (Fig. 3c, top panel). Following quantitative analysis of identical immunoblot membrane areas normalized as a function of total β-actin staining in corresponding fractions, MAG-EPA and MAG-DPA treatment significantly reduced TNFα and COX-2/β-actin staining density ratios when compared to the ratios quantified in untreated CFA rats (Fig. 3c, lower panels).

### Effect of MAG-ω3 treatment on metalloproteinase expression

**Fig. 4:**
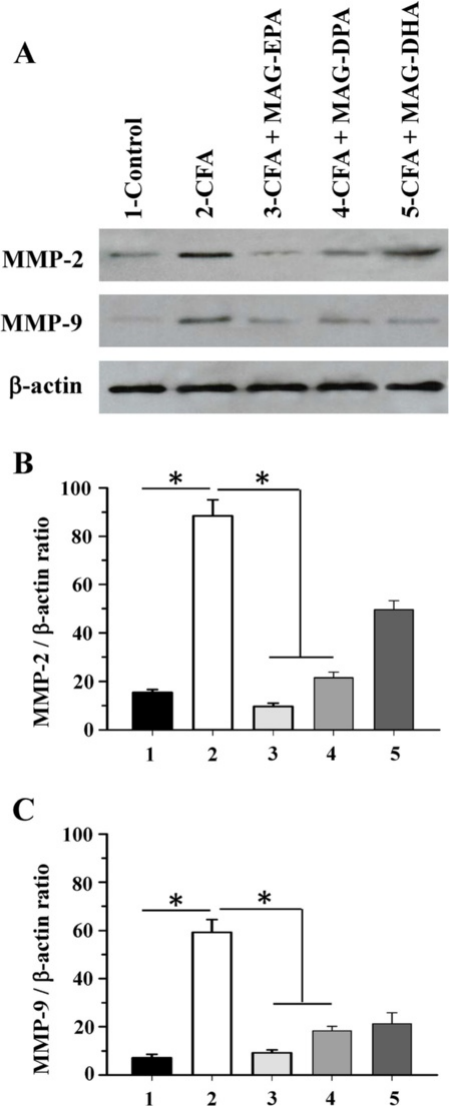
Effects of MAG-ω3 on metalloproteinase expression in paw tissues. **a** Western blot analysis of hind paw homogenates derived from control, complete Freund’s adjuvant (CFA), CFA + eicosapentaenoic acid monoglyceride (MAG-EPA)-, CFA + docosapentaenoic acid monoglyceride (MAG-DPA)-, and CFA + docosahexaenoic acid monoglyceride (MAG-DHA) -treated rats, using specific antibodies against matrix metalloproteinase (MMP)-2, MMP-9 and β-actin. **b** Quantitative analysis of MMP-2/β-actin and **c** MMP-9/β-actin density ratios as a function of experimental conditions (n = 6, *P <0.05)

Further experiments were performed to assess the effect of MAG-DHA, MAG-EPA and MAG-DPA treatments on MMP-2 and MMP-9 expression levels as these proteins have been shown to be involved in the degradation of joint cartilage. Western blot analyses were performed on hind paw and knee cartilage homogenates derived from control, CFA, CFA + MAG-DHA-, CFA + MAG-EPA- and CFA + MAG-DPA-treated rats (Fig. 4). Analysis revealed increased MMP-2 and MMP-9 expression levels in cartilage homogenates derived from CFA rats comparatively to controls. However, a reduced staining of MMP-2 and MMP-9 proteins expression was obtained following treatments with MAG-DHA, MAG-EPA and MAG-DPA compounds in CFA rats (Fig. 4a-c). β-actin staining remained constant from one preparation to the other (Fig. 4a).

### Effect of vitamin E on MAG-EPA-mediated anti-inflammatory properties

**Fig. 5:**
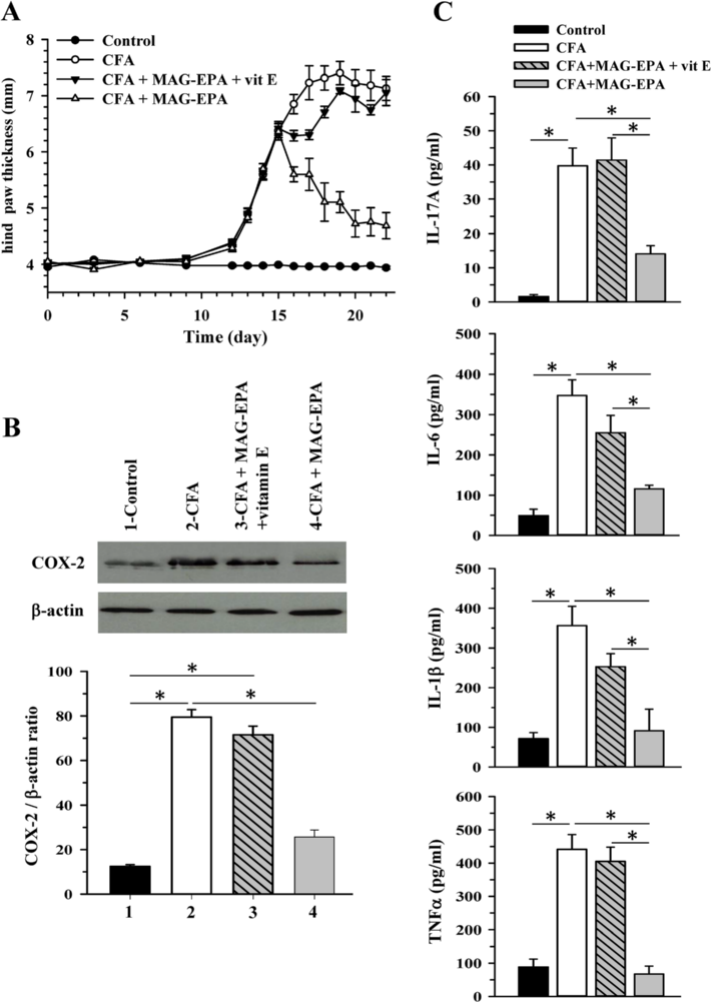
Effect of vitamin E treatment on eicosapentaenoic acid monoglyceride (MAG-EPA)-induced anti-inflammatory effects in complete Freund’s adjuvant (CFA) rats. **a** Hind paw thickness (mm) as a function of time (days) was measured in control, CFA, CFA + MAG-EPA-treated rats in the absence and presence of vitamin E (vit E). Results represent the mean ± standard error of the mean (n = 6 per group). **b** Typical western blots and subsequent quantitative analysis of paw homogenate fractions derived from control, CFA, CFA + MAG-EPA + vitamin E- and CFA+ MAG-EPA-treated rats using specific antibodies against cyclooxygenase (COX)-2 and β-actin. Staining densities in homogenates are expressed as a function of β-actin signals. (n = 6, *P <0.05). **c** Determination levels of IL-17A, IL-6, IL-1β and TNFα in plasma of control, CFA-treated, CFA + MAG-EPA-treated rats in the absence and presence of vitamin E, (n = 6, *P ≤0.05)

Additional experiments were performed to determine whether vitamin E (commonly used as an antioxidant in omega-3 formulations) exerts synergistic or antagonistic effects on anti-inflammatory properties of MAG-EPA in CFA animals. Combined treatment of vitamin E (53 mg/kg) with MAG-EPA (318 mg/kg) was administrated daily 15 days following CFA injection after which its effects on hind paw thickness, COX-2 expression levels and pro-inflammatory cytokine profiles were investigated. Figure 5a demonstrates that combined treatment of MAG-EPA and vitamin E in CFA rats incurred an increase in hind paw thickness similar to that of CFA-untreated animals. However, treatments with MAG-EPA alone significantly reduced hind paw thickness when compared to untreated CFA rats. The effect of combined MAG-EPA and vitamin E treatment was also assessed on COX2 protein expression by western blot in the different preparations of hind paw homogenates. Figure 5b shows that combined treatment of MAG-EPA and vitamin E inhibited the reduction in COX2 protein expression level observed in CFA + MAG-EPA treated animals. Lastly, the levels of the pro-inflammatory cytokines IL-17A, IL-1β, IL-6 and TNFα were determined in plasma derived from control, CFA- and CFA + MAG-EPA-treated rats in the absence and presence of vitamin E (Fig. 5c). Data demonstrate that combined MAG-EPA and vitamin E treatment also curtailed the effect induced by MAG-EPA on pro-inflammatory cytokine levels in CFA rats (Fig. 5c). Moreover, no significant difference was observed between CFA + MAG-EPA + vitamin E-treated and CFA-untreated rats (Fig. 5c). Taken together, these results indicate that vitamin E displays antagonistic effects on MAG-EPA anti-inflammatory properties in our rat model of RA.

## Discussion

The present study shows an anti-inflammatory effect of MAG-ω3 compounds in a rat model of CFA-induced arthritis. The ability of ω3 fatty acids to downregulate several aspects of inflammation suggests that these fatty acids may be important in determining the development and severity of inflammatory diseases and that they may be useful as a component of therapy. Particular interest in the therapeutic potential of ω3 fatty acids in RA was shown quite early on, because of the recognition that these fatty acids target arachidonic acid metabolism known to be involved in this disease. In the present study, we used a CFA model of RA to assess the effects of MAG-EPA, MAG-DPA and MAG-DHA treatment on disease progression and severity. MAG-EPA treatment (human equivalent of 3g/day) during 7 days was found to decrease paw swelling, pro-inflammatory marker levels (IL-17A, IL-6, IL-1β, TNFα, COX-2 and MMPs) as well as disease severity. We propose that MAG-EPA is able to reduce arthritis severity in a CFA rat model.

### Anti-inflammatory effects of MAG-ω3 compounds on arthritis severity

Clinical reports have shown that intake of ω3 PUFAs is associated with a reduction in the severity of RA [3, 26]. In the majority of these studies however, a heterogeneous mixture of the two main active ω3 PUFAs was used: EPA and DHA [26, 34]. Many studies have also reported differential effects of EPA, DHA and their metabolites both in a clinical setting and at the laboratory bench.

There are multiple factors that contribute to the differential effects of EPA and DHA, including differences in direct and indirect activation of transcription factors, impact of length, degree of saturation and stability of fatty acids on their efficacy, and differential efficiency for incorporation of the fatty acids into phospholipids [35]. Potency of the metabolites of EPA and DHA are often markedly different to the parent long-chain ω3 PUFA, and divergence in the effectiveness of enzymes to metabolize EPA and DHA can contribute to the observed diversity in cellular response [35]. A preclinical study demonstrated that both EPA and DHA suppressed streptococcal cell wall-induced arthritis in rats, with EPA being the more effective of the two fatty acids [36]. However, a second study showed rats fed an EPA-enriched diet had an increased incidence of arthritis in a collagen-induced arthritis (CIA) model [37]. Olson *et al*. demonstrated that dietary supplementation with DHA, but not with fish oil or DHA/EPA, significantly reduced arthritis severity, anti-collagen antibody production and inflammation associated with CIA in mice [38]. A recent study by Torres-Guzman *et al*. has shown an antinociceptive and anti-inflammatory effect of DHA following repeated systemic or intra-articular treatment in a mouse model of chronic CFA-induced knee arthritis [39]. To our knowledge, the biochemical effects of ω3 DPA have not been extensively studied in preclinical models due to the limited availability and high cost of pure compound.

In the present study, we assessed the ability of MAG-EPA, MAG-DHA and MAG-DPA to resolve inflammation in an *in vivo* model of RA induced by CFA. Fatty acids in monoglyceride form confer increased bioavailability of ω3 and are generally recognized as safe and are widely used as emulsifying agent in the food industry. In a previous study, we have demonstrated that DHA monoacylglyceride increased the systemic bioavailability of DHA compared to commercially available marine oil [30–32]. Omega-3 monoglycerides have better solubility in physiological solution and pharmacokinetics than omega-3 methyl ester or ethyl ester (EE) and more stable than omega-3 free fatty acid. Preclinical and clinical studies showed that the intestinal absorption of DHA and EPA given as EE was lower than seen in the case of triglyceride (TG) or free acid [40, 41]. Moreover, Dyerberg *et al*. and Cruz-Hernendez *et al*. have shown that EPA in the form of monoacylglyceride alone or in re-esterified TGs increases the oral bioavailability of EPA compared to natural TG form made from fish oil [40, 42]. Moreover, Banno *et al*. demonstrated that DHA monoglycerides and diglyceride are absorbed and transported more effectively than DHA-TG and EE in rats under a water-restricted condition [43]. In a preclinical model, Cruz-Hernandez *et al*. have shown that malabsorption due to enzyme insufficiency may lead to decreased circulating and tissue levels of EPA and such a deficiency can be reversed using MAG provided as sn-1(3)-MAG or protected sn-2-MAG [42]. Hence, Philippoussi *et al*. demonstrated that lipids in monoglyceride form display better induction of apoptosis in T-cells when compared with corresponding free fatty acid [44]. According to these data, we thought that the MAG-ω3 might be favorable in terms of absorption and utilization efficiency. Herein, our data revealed that MAG-EPA and MAG-DPA treatments were more effective than MAG-DHA in resolving inflammation and reducing arthritis severity in our preclinical model of RA. Histopathological analysis also correlated with the reduction in clinical scores, showing an overall reduction in both inflammation and bone and cartilage destruction in joints of animals treated with MAG-EPA or MAG-DPA.

Current literature indicates that supplemental ω3 PUFA decreases inflammatory cytokines [16] and eicosanoids [17, 38] in patients with RA. As a result, these effects should reduce pain and cartilage destruction which, in turn, may lead patients to decrease their use of pain-controlling drugs. Randomized controlled trials of ω3 fatty acids (at doses between 1 and 7g per day) in RA have reported improvements in several clinical outcomes including reduced duration of morning stiffness, reduced number of tender or swollen joints, reduced joint pain, reduced time to fatigue, increased grip strength and decreased use of pain-controlling drugs [3, 18, 45]. In a recent meta-analysis encompassing data from 17 trials on ω3 fatty acids and pain [26], fish oil was found to reduce patient-assessed joint pain, duration of morning stiffness, number of tender joints and use of pain-controlling drugs. It is therefore of key clinical interest to find an easy-to-use, well-absorbed ω3 PUFA that exerts a pro-resolving effect and consequently with the ability to reduce arthritis severity and progression.

### Possible mechanisms underlying the anti-inflammatory effect of MAG-EPA and MAG-DPA in the current CFA model of arthritis

Although several studies have demonstrated anti-inflammatory effects of ω3 PUFA [3, 18], less is known as to the molecular mechanisms underlying these effects. One possible explanation, based on current literature, is that both direct and indirect effects may explain the biological actions of ω3 PUFA. Direct effects may include competition of DHA, EPA or DPA with arachidonic acid as a substrate for COX and LOX, thus reducing the production of inflammatory eicosanoids [3, 46–48]. Moreover, we and others have recently demonstrated that the anti-inflammatory actions of ω3 PUFA and MAG-ω3 compounds may partially be explained by inhibition of NFκB-mediated COX-2 induction and activity [11, 49]. Expression of both COX-1 and COX-2 is increased in the synovium of patients with RA [11]; in addition, synovial fluid contains high levels of pro-inflammatory eicosanoid products from both the COX and LOX pathways [11] as well as high levels of pro-inflammatory cytokines including TNF, IL-1β, IL-17A, IL-6, IL-8 and GMCFS [50]. In this study, we demonstrate that MAG-EPA treatment is able to reduce the NFκB and p38 MAPK activation pathways, resulting in decreased levels of pro-inflammatory mediators such as COX-2, IL-17A, TNFα, IL-6, IL-1β, MMP-2 and MMP-9.

Indirect effects may also contribute to the biological actions of ω3 PUFA, including the participation of Rvs and protectins, which are lipid mediators enzymatically synthesized *in vivo* from EPA, DPA or DHA and shown to promote the resolution of inflammation with greater potency than their parent precursors [14, 51]. To date, there are only limited data investigating the actions of Rvs in animal models of arthritis. Accordingly, protective effects of aspirin-triggered-RvD1 (AT-RvD1) were observed following an intraplantar injection of CFA as a model of inflammatory arthritic pain [52]. In this latter study, AT-RvD1 (100 ng intraperitoneally twice daily) was shown to have antihyperalgesic effects, reducing hind paw withdrawal frequency, which was associated with decreased TNFα and IL-1β within the paw [52]. Moreover, several Rvs including RvD1, RvD2 and RvE1 have recently been identified as potent analgesics for treating inflammatory pain, acting as potent endogenous inhibitors that differentially regulate transient receptor potential subtype V1 (TRPV1) and A1 (TRPA1) agonist-elicited acute pain [14, 51].

RA is also known to be directly associated with an increase in ω-6 and a reduction in ω-3 fatty acid levels in blood circulation and tissues [3]. Previous studies from our group have shown that MAG-ω3 treatment increases the level of ω3 PUFA in plasma, red blood cells and tissues, suggesting a high ω3 PUFA bioavailability and thereby likely contributing to reducing inflammation [30–32]. We also established that MAG-ω3 compounds were metabolized by lipoxygenases and CYP450 to generate metabolites mediating anti-inflammatory effects in our experimental models [30–32]. We propose that MAG-EPA not only improves the plasma and cell/tissue content of EPA but also increases the production of beneficial metabolites such as Rvs [14] and exerts pro-resolving actions in our model of RA. Accordingly, elevated levels of 5-LO and 15-LO (key enzymes involved in Rv and lipoxin biosynthesis) have also been detected in RA synovium [53, 54]. A study using mass spectrometry-based lipidomic analyses has identified RvD5 and Maresin 1(MaR1) within the synovial fluid from RA patients [55]. Such findings warrant further study, such as stratifying patients according to disease severity, and taking into account differences in therapeutic and dietary intervention with ω3 supplementation.

Hypotheses would suggest that vitamin E and ω3 PUFAs have synergistic anti-inflammatory effects [56]. However, vitamin E is a potent antioxidant interrupting lipid peroxidation that has been purported to have antagonistic effects to ω3 PUFA, in particular in carcinogenesis [57]. Our results corroborate the hypothesis of a converse interaction between ω3 PUFA and vitamin E intake on inflammatory biomarkers. PUFAs are able to auto-oxidize such that their incorporation has been found to induce lipid peroxidation and apoptosis *in vitro* and in animal models of cancer [58, 59]. In these latter studies, ω3 PUFAs led to tumor growth suppression while adding vitamin E to ω3 PUFAs abolished this effect [58, 59], suggesting that ω3 PUFA-induced lipid peroxidation is likely not toxic per se but rather acts as a tumor cell growth and apoptosis regulator [60]. Moreover, in lung cells, antioxidants have been demonstrated to increase tumor cell proliferation *in vitro* and *in vivo* by reducing p53 activation [61]. Experimental studies and large clinical trials quite convincingly suggest that antioxidants, including isoflavones, carotenes and vitamins, should not be recommended for the prevention of lung cancer and that their use may promote tumor growth [62–64]. Moreover, an epidemiological study by Julia *et al*. demonstrated an inverse relationship between PUFA intake and elevated levels of CRP in individuals taking vitamin E supplements [65]. Such interaction between PUFAs and vitamin E intake in inflammation will clearly necessitate further investigation in preclinical models of RA and human studies.

## Conclusion

The present findings demonstrate that MAG-EPA, without vitamin E, exerts anti-inflammatory properties in a CFA animal model of arthritis. Furthermore, when administrated *per os*, MAG-EPA represents a stable compound which could serve as a precursor to generate a variety of PUFA-derived mediators such as Rvs known to directly mediate anti-inflammatory and pro-resolving effects through specific receptors. Consequently MAG-EPA formulations without vitamin E could provide a new and interesting approach for the management of rheumatoid arthritis diseases.

## Abbreviations

AT-RvD1: aspirin-triggered-resolvin D1
BSA: bovine serum albumin
CFA: complete Freund’s adjuvant
CIA: collagen-induced arthritis
COX-2: cyclooxygenase-2
CRP: C-reactive protein
CYP450: cytochrome P450
EE: ethyl ester
ELISA: enzyme-linked immunosorbent assay
GI: gastrointestinal
GMCFS: granulocyte/macrophage colony stimulating factor
IL-17A: interleukin 17A
IL-1β: interleukin 1β
IL-6: interleukin 6
LOX: lipoxygenase
LTB4: leukotriene B4
MAG-DHA: docosahexaenoic acid monoglyceride
MAG-DPA: docosapentaenoic acid monoglyceride
MAG-EPA: eicosapentaenoic acid monoglyceride
MaR1: maresin 1
MMP: matrix metalloproteinase
NFκB: nuclear factor kappa B
NSAID: nonsteroidal anti-inflammatory drug
p38 MAPK: p38 mitogen-activated protein kinase
PD1: protectin D1
PGE2: prostaglandin E2
PUFA: polyunsaturated fatty acid
RA: rheumatoid arthritis
RvD1: resolvin D1
RvD2: resolvin D2
RvD5: resolvin D5
RvE1: resolvin E1
SEM: standard error of the mean
TBS: Tris-buffered saline
TG: triglyceride
TNFα: tumor necrosis factor α
